# Implantation of Recyclability and Healability into Cross-Linked Commercial Polymers by Applying the Vitrimer Concept

**DOI:** 10.3390/polym12061322

**Published:** 2020-06-10

**Authors:** Mikihiro Hayashi

**Affiliations:** Department of Life Science and Applied Chemistry, Graduated School of Engineering, Nagoya Institute of Technology, Gokiso-cho, Showa-ku, Nagoya, Aichi 466-8555, Japan; hayashi.mikihiro@nitech.ac.jp; Tel.: +81-52-735-7159

**Keywords:** vitrimers, commercial polymers, healability: recyclability: practical application

## Abstract

Vitrimers are a new class of cross-linked materials that are capable of network topology alternation through the associative dynamic bond-exchange mechanism, which has recently been invented to solve the problem of conventional cross-linked materials, such as poor recyclability and healability. Thus far, the concept of vitrimers has been applied to various commercial polymers, e.g., polyesters, polylactides, polycarbonates, polydimethylsiloxanes, polydienes, polyurethanes, polyolefins, poly(meth)acrylates, and polystyrenes, by utilizing different compatible bond-exchange reactions. In this review article, the concept of vitrimers is described by clarifying the difference from thermoplastics and supramolecular systems; in addition, the term “associative bond-exchange” in vitrimers is explained by comparison with the “dissociative” term. Several useful functions attained by the vitrimer concept (including recyclability and healability) are demonstrated, and recent molecular designs of vitrimers are classified into groups depending on the types of molecular frameworks. This review specifically focuses on the vitrimer molecular designs with commercial polymer-based frameworks, which provide useful hints for the practical application of the vitrimer concept.

## 1. Introduction

Cross-linking is a method used to link polymers to form a 3D network. Various physical properties of polymeric materials (e.g., thermal/mechanical properties and solvent resistance) can be modified or enhanced by cross-linking. The industrial use of the cross-linking process arises from the vulcanization of rubbers. The vulcanization of rubbers was discovered by C. Goodyear, and later the detailed mechanism of the cross-linking formation was clarified by T. Hancock in the mid-19th century [1]. Since the discovery of vulcanization, cross-linked polymeric materials have been adapted in industrial societies and used in our lives (e.g., tires, shoes, shock absorbers, adhesives, and contact lenses). However, there are some issues that have to be solved to adjust the materials to modern society. The biggest defects of these conventional cross-linked materials are the poor recyclability and lack of healabilty.

The defects are attributed to the irreversible nature of cross-link points. In conventional cross-linked materials, the cross-linking reaction progresses through the permanent covalent bond linking of polymer chains. Thus, cross-linking is an irreversible process, and cross-linked materials cannot be melted and dissolved in any solvents once cross-linking has occurred. Currently, the sustainability of materials is an important issue owing to the limitation of petroleum resources and environmental pollution, which makes it essential to develop recyclable and healable cross-linked materials.

**Figure 1:**
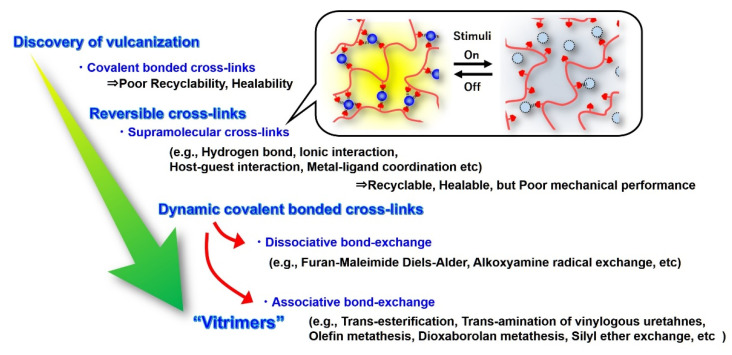
Evolution of cross-linked materials from the discovery of vulcanization to vitrimers.

To solve the problem related to the recyclability and healability of conventional cross-linked materials, the use of reversible cross-links, instead of irreversible cross-links, has been promoted. The evolution of cross-linked materials, from the discovery of vulcanization to vitrimers, is summarized in Figure 1. Since the early 2000s, supramolecular bonds (e.g., hydrogen bonds, ionic association, host–guest interactions, metal–ligand coordination, and π–π interactions) have been introduced to construct the polymeric network structure [2,3,4,5]. The term “supramolecular” is associated with “supramolecular chemistry,” which was proposed by J-M. Lehn [6,7]. Because supramolecular cross-links have a reversible nature, the network structure constructed from supramolecular cross-links can be collapsed and re-constructed under the switching on/off of external stimuli, such as heat and UV light. Therefore, supramolecular cross-linked materials can be melted and become fluid, which enables recyclability and healability. Academically, there have been many molecular designs that have achieved an efficient self-healing ability and recyclability on the basis of the reversible nature of supramolecular cross-links. However, the material strength of supramolecular cross-linked materials is generally much weaker than those of materials with covalently bonded cross-links. Poor material strength has limited the industrial application of the concept of supramolecular cross-links, although the combination of such weak physical bonds with stable covalently bonded cross-links has considerable potential to enhance material toughness, in which the weak bonds can behave as sacrificial bonds under the application of stress [8,9,10,11,12,13].

More recently, on the basis of the abovementioned background, a special type of covalent bond has been developed with the capability of dissociation and re-association under external stimuli. Such a type of covalent bond is termed as a dynamic covalent bond, according to the dynamic nature of the bond. The networks constructed from such dynamic covalent bonded cross-links are referred to as covalent adaptive networks (CANs) [14,15,16]. The materials with CANs possess a sufficient material strength because the dynamic exchange of the cross-links is frozen in the absence of external stimuli, whereas the materials show recyclability and healability under the application of external stimuli, such as heat and UV light, owing to the activation of bond exchange. In the recent studies on dynamic covalent bonds, dynamic covalent bonds are classified into two groups depending on the bond exchange mechanism [17,18,19], which will be explained in more detail in the next section. One group is the “dissociative” dynamic covalent bond, in which bond exchange undergoes distinct separate steps of dissociation and re-association under the switching on/off of the stimuli. The most classical examples of dissociative dynamic bond may be the Diels–Alder reaction or the reaction of alkoxyamine, which have been pioneeringly incorporated into cross-linked polymer networks by Wudl et al. and Otsuka et al., respectively [20,21]. The other group is the “associative” dynamic covalent bond, which was invented much later than the dissociative dynamic covalent bond. In 2005, Bowman et al. reported the preparation of novel cross-linked materials that exhibited useful functions, such as plasticity and shape changes, without residual stress [22]. These functions were achieved by the incorporation of exchangeable bonds into the network on the basis of radical addition–fragmentation upon exposure to light. More recently, L. Leibler et al. constructed the concept of vitrimers, where the mechanism of bond exchange via trans-esterification has been introduced into the network structure in their first series of vitrimer studies [23,24,25]. The abovementioned examples are representative pioneering works on the associative dynamic covalent bond. Unlike dissociative dynamic covalent bonds, the bond exchange in associative dynamic covalent bonds occurs without distinct separated steps of bond dissociation and re-association. Thus, bond dissociation and re-association can progress simultaneously. The materials constructed from an associative dynamic covalent bond are termed “vitrimers,” and many molecular designs with unique useful functions, such as recyclability and healability, have been under development.

In this review article, the general characteristics of vitrimeric properties and functions are described, during which the strict definition of vitrimers is also noted. The implantation of the concept of vitrimers into commercial polymers, such as polyesters, polylactides, polycarbonates, polydimethylsiloxanes, polydienes, polyurethanes, polyolefins, poly(meth)acrylates, polystyrenes, etc., have been so far explored to realize the practical application, and thus, in the later part, the molecular designs based on these commercial polymer frameworks are summarized. As far as the author knows, this is the first review article of vitrimers that are specifically designed based on the frameworks of commercial polymers, which can give some hints to the practical application of the vitrimer concept.

## 2. Characteristics of the Bond Exchange Mechanism of Vitrimers

**Figure 2:**
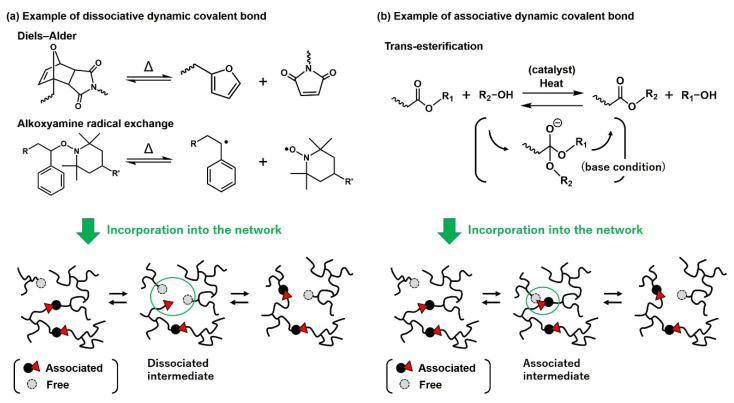
Examples of (**a**) dissociative dynamic covalent bonds and (**b**) associative dynamic covalent bonds, and the schematic for the difference in the bond exchange mechanism in the network.

First, the characteristics of the vitrimer bond exchange mechanism are explained. Figure 2 shows some examples of the bond exchange mechanism for dissociative and associative dynamic covalent bonds. As the first example of dissociative dynamic covalent bond (Figure 2a), in the Diels–Alder reaction between furan and maleimide groups, the bond is dissociated under heating, and the dissociated bond is re-associated via the retro Diels–Alder reaction by the removal of heat stimuli. Similarly, for alkoxyamine, radical decoupling occurs under heating, which shifts the chemical equilibration to the dissociated free state, and equilibration is again shifted to the associated state by the removal of heat. In both examples, bond exchange occurs by the switching on/off of the heat, which provides recyclability and healability to the materials. In this respect, the bond exchange mechanism of the dissociative dynamic covalent bond is very similar to that of thermoplastic elastomers/gels and supramolecular cross-linked materials, in which the weak physically bonded cross-links are gradually dissociated under heating.

In Figure 2b, the trans-esterification reaction is schematically demonstrated as an example of the associative dynamic covalent bond. In this reaction, the R group is replaced between different components when ester and alcohol are reacted. In usual cases, acid or base catalysts are required for the efficient exchange progress. Bond dissociation and re-association occur simultaneously without undergoing the distinct dissociation step; thus, trans-esterification is classified as an associative dynamic covalent bond. In Figure 2b, the vitrimer network with trans-esterification-based bond exchange is also illustrated, where the ester bond (associated) and OH (free) group are introduced in the network structure. By heating in the presence of catalysts, the free OH group and ester bond exchange the pair, which continuously occurs at temperatures higher than the activation temperature of the bond exchange. The biggest characteristic for the network with the associative bond exchange mechanism is that the network integrity is maintained, whereas the network topology is constantly altered during bond exchanges. In other words, the cross-link density remains constant during the bond exchanges, which differs from the bond exchange for the network with a dissociative bond exchange mechanism.

**Figure 3:**
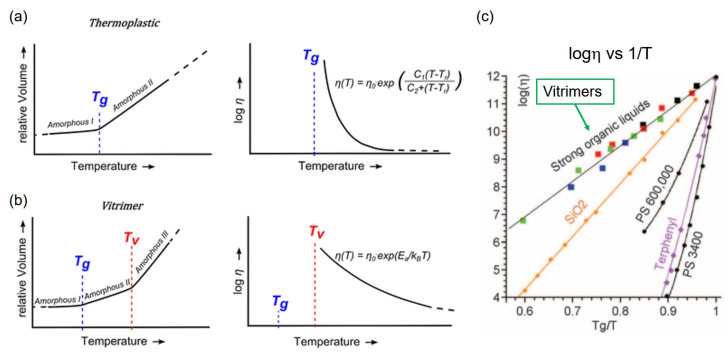
Correlation chart of relative volume vs. temperature and viscosity vs. temperature for (**a**) thermoplastics and (**b**) vitrimers. Reprinted with permission from ref. [24]. Copyright (2012) American Chemical Society. (**c**) Variation of viscosity as a function of inverse temperature according to ref. [23], where the temperature is normalized to 1 at the glass transition temperature (*T*_g_). The plots of vitrimer samples are for epoxy/anhydride with 5% (red squares) and 10% (black squares) zinc acetylacetonate Zn(acac)_2_; for epoxy/acid with 5% (green squares) and 10% (blue squares) zinc acetate Zn(ac)_2_. The other plots are for silica (SiO_2_), polystyrene (*M*~600,000 and 3400), and terphenyl. From ref. [23] (Montarnal, D.; Capelot, M.; Tournilhac, F.; Leibler, L., *Science* 334 (6058), 965–968, (2011)). Adapted with permission from AAAS.

On the basis of the abovementioned features of the bond exchange mechanism, the correlations of temperature–relative volume and temperature–viscosity are demonstrated in Figure 3a,b, according to the report by L. Leibler et al. [24]. For thermoplastic materials (Figure 3a), upon heating, the volume expansivity suddenly increases at the glass transition temperature, above which the materials are in a molten state. An increase in the volume expansivity is due to the activation of segmental mobility, which results in the change of the free volume of the chains. At temperatures above *T*_g_, the correlation of temperature–viscosity follows the Williams–Landel–Ferry (WLF) equation, where the dependence of the decrease in viscosity on temperature is steep. For supramolecular materials and materials with dissociative dynamic covalent bonds, the viscosity drop at temperatures above the dissociation temperature of the dynamic covalent bond also follows the WLF equation. On the other hand, for vitrimer materials (Figure 3b), the dependence of relative volume on temperature changes at two different temperatures; the first change of the dependency is due to the glass transition, and the other change at a higher temperature is due to the activation of the bond exchange. Here, the temperature of the bond exchange is tentatively defined as the vitrimer temperature, *T*_V_. The dependence of the decrease in viscosity on temperature at temperatures above *T*_V_ does not follow the WLF equation, but the Arrhenius-type equation. This dependence is observed because the decrease in viscosity relies not only on the expansion of the free volume of the chain, but mainly on the bond exchange rate. The Arrhenius-type dependence of viscosity is maintained when the temperature is far above *T*_V_, whereas the dependence of viscosity is switched to the WLF-type when the temperature approaches *T*_g_ [19]. Specifically, for vitrimers, the dependence of the decrease in viscosity upon heating at *T* >> *T*_V_ is considerably milder compared with that of thermoplastics. In fact, the correlation of temperature–viscosity for silica glasses follows the Arrhenius-type equation (Figure 3c, see the plot of SiO_2_); thus, vitrimers are known to have unique reprocessability that is comparable to melted glass-blowing; for silica glasses, the shape can be re-processed by heating, in which glasses do not show a sudden flow, even though they are molten. Similarly, vitrimers can be re-processed without a sudden flow, which enables re-shaping into complex shapes without the need for molds. Such similarities with vitreous silica materials are, indeed, the origin of the generic name, vitrimers.

## 3. Characteristics of the Physical Properties of Vitrimers

**Figure 4:**
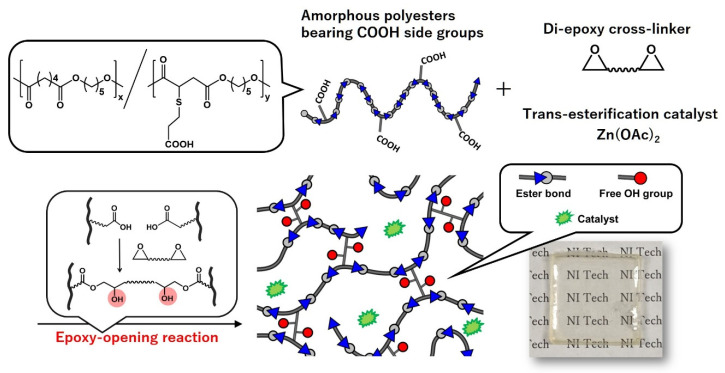
Molecular design of amorphous low *T*_g_ polyester-based vitrimers reported by M. Hayashi et al. from ref. [26]. Reproduced/adapted by permission of The Royal Society of Chemistry.

In this section, the basic physical properties of vitrimers are demonstrated on the basis of the report from M. Hayashi et al. for trans-esterification-based vitrimers (see the molecular design in Figure 4) [26]. In their report, vitrimer samples were prepared by cross-linking the low *T*_g_ amorphous polyesters bearing multiple COOH side groups (PE-COOH) with di-epoxy cross-linkers. PE-COOH had an average molecular weight (*M*_n_) of 17 kg/mol and 24 COOH groups per chain. PE-COOH was blended with 1,4-butanediol diglycidyl ether in the presence of the trans-esterification catalyst, zinc acetate (Zn(OAc)_2_), by the solution casting method, where the mole ratio of Zn(OAc)_2_ to COOH groups was set to be 20 mol%. After drying, the cross-linking reaction between epoxy and COOH groups was carried out by heating at 120 °C for 4 h, which resulted in a homogenous cross-linked film. In this design, OH groups were generated by the epoxy opening reaction. Thus, the final network possessed OH groups and ester bonds, which provided the material with the bond exchange capability through trans-esterification at a high temperature in the presence of catalysts. The *T*_g_ of the vitrimer film was −40 °C, and the decomposition temperature (*T*_d_) was much higher than 200 °C. As a control sample, a cross-linked film without any trans-esterification catalysts was also prepared.

**Figure 5:**
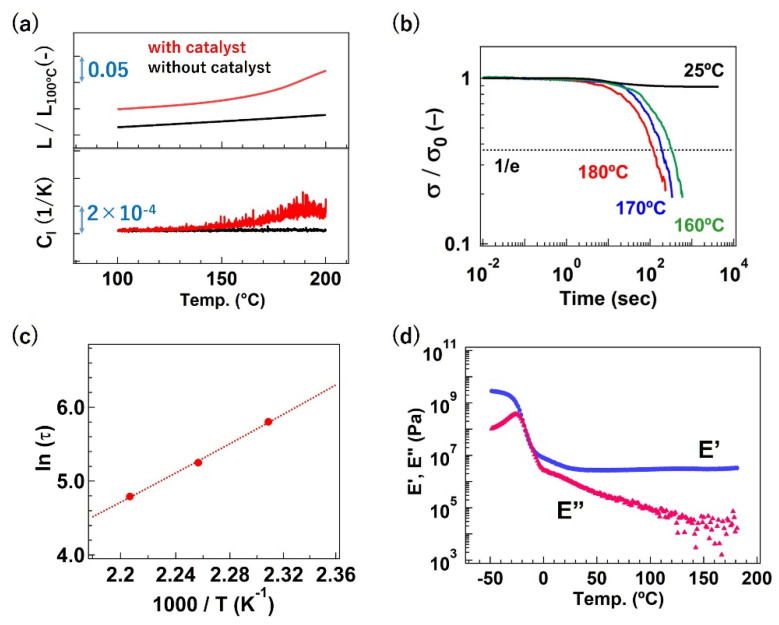
(**a**) Dilatometry data, (**b**) stress–relaxation, (**c**) Arrhenius plots of *τ*, and (**d**) rheology data with the temperature ramp mode for the amorphous low *T*_g_ polyester-based vitrimers reported by M. Hayashi et al. In (**a**), the y-axis of *L*/*L*_100°C_ represents the sample length normalized by the length at 100 °C, whereas the y-axis of *C*_l_ represents the linear expansion coefficient. In (**b**), the y-axis indicates the stress (*σ*) normalized by the initial stress (*σ*_0_). In (**d**), *E′* and *E″* represent the storage modulus and loss modulus, respectively. From ref. [26]. Reproduced/adapted by permission of The Royal Society of Chemistry.

Conventionally, for vitrimers, the bond exchange at high temperatures can be confirmed by performing dilatometry and stress–relaxation tests. Occasionally, a creep test is also measured to confirm the bond exchange. Figure 5a represents the dilatometry data for the abovementioned vitrimer samples with and without Zn(OAc)_2_. The changes in sample length and in the calculated linear expansion coefficient (*C*_l_) are plotted as a function of temperature (the temperature ramp rate was 3 °C/min). The sample without Zn(OAc)_2_ showed a constant change in sample length, whereas the deviation from the constant sample length change, i.e., softening, was observed at approximately 140 °C for the sample with Zn(OAc)_2_. Note that *T*_g_ and *T*_d_ are far from the temperature range of this softening. On the basis of the difference between the two samples, the deviation from the constant sample length indicated the activation of the bond exchange via trans-esterification. In addition, the bond exchange feature was clearly reflected in the stress–relaxation behavior, as shown in Figure 5b, for the sample with Zn(OAc)_2_. At 25 °C, stress–relaxation was not observed, which means that the bond exchange was frozen. At a temperature above the softening temperature (ca. 140 °C), a great stress–relaxation was observed, and the relaxation rate increased with an increase in the temperature. This suggests that the bond exchange rate was increased with an increase in the temperature.

The relaxation time *τ* can be typically defined at *t* when the stress normalized by the initial stress decays to 1/e, under the assumption of the Maxwell-type relaxation. Note that, in some recent reports, the stress–relaxation behaviors of vitrimers were not described by the simple Maxwell model, whereas these were well described by the stretched exponential function based on the Kohlrausch–Williams–Watts (KWW) equation by assuming a certain distribution of relaxation time [27,28]. The values of ln *τ* were plotted as a function of inverse temperatures in Figure 5c. The linear relationship is shown in the plot, which indicates that the relationship between the temperature and *τ* follows the Arrhenius-type function, $\tau =\tau_{0}exp\left(\frac{E_{a}}{RT}\right)$, where *R*, *T*, and *τ*_0_ are the gas constant, measurement temperature, and pre-exponential factor, respectively. The activation energy of the bond exchange, *E*_a_, can be estimated from the slope of the linear approximation line in the plot; the obtained value of *E*_a_ is ca. 83 kJ/mol. Note that the *E*_a_ estimated by the Arrhenius equation for vitrimers is not necessarily identical to the value estimated from the model reaction for the same reactive pairs using small molecules; this is because some effects of the chain restriction, due to the cross-linking, contributes to the *E*_a_ value for vitrimer materials.

The Arrhenius relation between *τ* and temperature is indeed the characteristic of the flow property of vitrimers. This indicates that stress–relaxation is governed by the progress of the associative bond exchange. Here, it should be remembered that, for true vitrimers, the cross-link density must be kept constant during the bond exchange. This fact can be confirmed by the rheological measurement with the temperature ramp mode. For the abovementioned vitrimer sample with Zn(OAc)_2_, the plateau modulus was kept constant, up to 200 °C (Figure 5d), although great stress–relaxation was observed at a temperature higher than the softening temperature (ca. 140 °C). This observation is the indication of the constant cross-link density. The constant cross-link density during the bond exchange is the strict definition of vitrimer materials.

**Figure 6:**
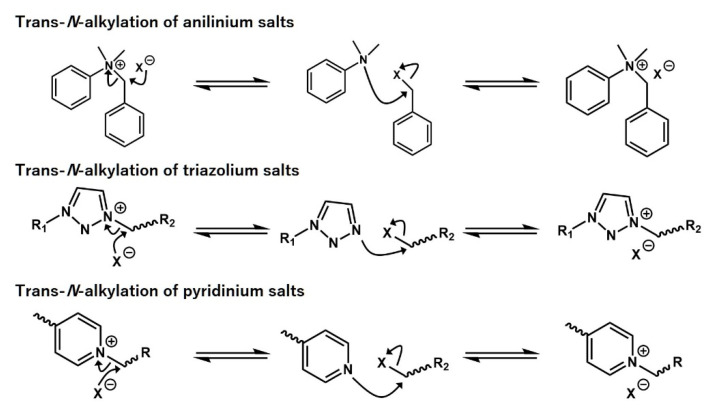
Reported examples of the trans-*N*-alkylation reaction of quaternized amino-based salts.

It should be also noted that there are some examples of dissociative bond exchangeable materials with vitrimeric characters; these materials show the stable rubbery plateau without a decrease in modulus, great stress–relaxation at high temperatures, and Arrhenius-type dependence of *τ*. The trans-*N*-alkylation reaction of quaternized amino-based salts, such as anilinium salts [29,30], triazolium salts [31,32,33], and pyridinium salts [34,35], is among the most well-known examples of such a dissociative bond exchange (Figure 6). In addition, the network, with the incorporation of hindered urea exchange [36,37,38] and oxime-enabled transcarbamoylation [39] were reported to show such vitrimer-like properties. In this case, these properties are the consequence of either fast bond reformation or the large equilibrium constant *K*_eq_ in favor of the associated state within high temperatures. Thus, eventually, the network integrity is well maintained, while the stress–relaxation is governed by the bond exchange. To distinguish them from true vitrimers, these materials are sometimes termed as vitrimer-like materials.

## 4. Representative Useful Functions of Vitrimers

**Figure 7:**
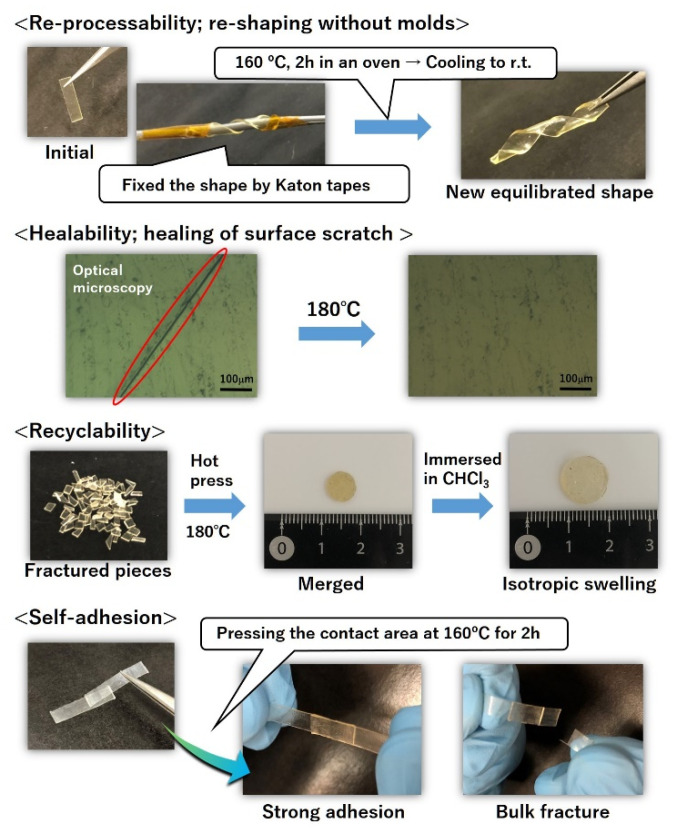
Representative functions of vitrimers, where the vitrimer sample was the amorphous low *T*_g_ polyester-based vitrimer reported by M. Hayashi et al. from ref. [26]. Reproduced/adapted by permission of The Royal Society of Chemistry.

Owing to the bond exchange property, vitrimers show useful functions that cannot be achieved in conventional cross-linked materials. In this section, the representative functions, such as re-processability, healability, recyclability, and self-adhesion, are introduced on the basis of the report by the author. Figure 7 shows the visual representation of these functions, where the vitrimer used was explained in the previous Section 3.

First, re-processability is one of the characteristic functions of vitrimers. The strip-shaped vitrimer film was wrapped in a metal bar and fixed by Kapton tape. The fixation was maintained at 160 °C for 2 h in an oven. After the cooling and removal of the tape, the spiral-shaped sample was obtained, and there were no signs of sample flowing on the film edge. Similarly, film thinning is possible by hot pressing at 160 °C. In conventional cross-linked materials, the cross-link points are formed by irreversible covalent bonds; thus, the materials cannot be re-processed once the cross-linking reaction is completed. For thermoplastics, re-processing is possible; however, the materials suddenly flow at higher temperatures. Therefore, complex shapes cannot be made without molds. On the other hand, vitrimers can be re-processed by heating the sample to a temperature above the activation temperature of the bond exchange, without the need for molds, because vitrimers do not show a sudden flow, owing to the associative bond exchange nature.

Healability and recyclability are also achieved by utilizing the vitrimer concept. These functions are highly important to meet the recent demand to realize a sustainable society, because materials with healability and recyclability can prolong the material’s life and reduce waste. Healability is shown in Figure 7. A film with a 0.5 mm deep scratch was heated at 180 °C. After a period of time, the scratch disappeared, as shown in the optical microscope image. This result suggests that the chains near the scratch can be reconfigured by the bond exchange at a higher temperature than the activation temperature of the bond exchange. To demonstrate recyclability, the film sample was first fractured into small pieces, and the fractured pieces were collected in a disk-shaped mold. After hot pressing at 180 °C, the fractured pieces were merged into one disk. The merged sample was immersed in chloroform, and the disk-shaped sample was then swollen isotropically without breaking it into pieces. This phenomenon indicates that the sufficient bond exchange and interpenetration of the chains progressed between the fractured pieces. In addition, the spectra of Fourier-transform infrared spectroscopy (FT-IR) for the 1st and 2nd recycled films were well overlapped with that of the flesh film, which indicates that the chemical structures before and after the recycling processes were maintained.

Self-adhesion is also an interesting function of vitrimers. A conventional cross-linked film does not show strong adhesion because of the poor network chain mobility. Therefore, to adhere the cross-linked films, an adhesive is needed, which causes problems, such as the degradation of health and the non-negligible distortion of the final products. On the other hand, the bond exchange of vitrimers enables strong self-adhesion, as shown in Figure 7. Two vitrimer films were overlapped, and a 50 g weight was placed on the overlapping part. The pair of films, with the weight, was placed in an oven at 160 °C for 2 h. After cooling and removing the weight, it was confirmed that the two films were well adhered (self-adhesion). Of course, the cross-linked films without Zn(OAc)_2_ did not show self-adhesion. The adhesion strength was tentatively evaluated by hanging the weight. The adhered films with an adhesion area of 4 mm × 4 mm could hold a 200 g weight, and the bulk fracture was observed when a 250 g weight was attached. The results indicate that the bond exchange progressed sufficiently between the different surfaces of the vitrimers, and new bonds were newly formed across the surfaces.

## 5. Recent Studies on the Implantation of the Vitrimer Concept into Commercial Polymers

As described in the above sections, vitrimers exhibit various useful functions, which are all due to the associative bond exchangeable nature. The vitrimer concept has been introduced into the framework of commercial polymers, which contributes to the application of the vitrimer concept into practical materials. Below, recent achievements of the implantation of the vitrimer concept into commercial polymer-based materials using various bond exchangeable mechanisms are summarized.

### 5.1. Polyesters

**Figure 8:**
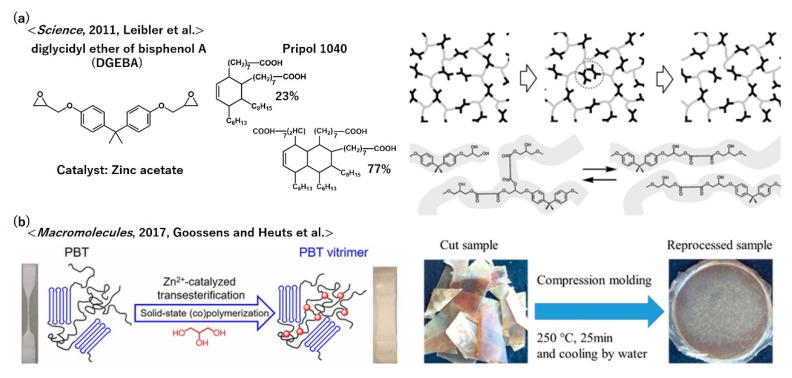
Examples of vitrimer polyesters according to (**a**) ref. [23] and (**b**) ref. [43]. (**a**) From (Montarnal, D.; Capelot, M.; Tournilhac, F.; Leibler, L., Science 334 (6058), 965–968, (2011)). Adapted with permission from AAAS. (**b**) Reprinted with permission from ref. [43]. Copyright (2017) American Chemical Society.

A pioneering series of vitrimer studies was carried out for polyesters by incorporating the trans-esterification bond exchange mechanism [23,24,25]. L. Leibler’s group first demonstrated a very simple design of polyester vitrimers, where the components were diglycidyl ether of bisphenol A (DGEBA = di-epoxy compound) and Pripol 1040 (a mixture of di-acid (23%) and tri-acid (77%)) (Figure 8a). These monomers were reacted in the presence of a trans-esterification catalyst, zinc acetate. The key for this design is the generation of OH groups in the network with ester groups through the epoxy-COOH reaction and the use of the trans-esterification catalyst, which enabled trans-esterification at high, but acceptable, temperatures. In their first report, the essence of vitrimers, such as softening without a sudden flow, great stress relaxation while showing the stable rubbery plateau, and the Arrhenius dependence of viscosity, has been demonstrated by highlighting the most important vitrimeric nature, i.e., the constant cross-link density during the bond exchange. Recyclability and re-processability have already been shown in their first report. Later, the bond exchange mechanism of the catalytic trans-esterification reaction was clarified by the same group [40], and the evolution of the network formation was later investigated in detail with statistical analysis on the basis of the fragment approach by Williams et al. [41]. These initial findings for vitrimers soon inspired other research groups to explore other various vitrimer molecular designs and functionalization. Indeed, Leibler’s group performed the vitrimerization of industrial polyesters, that is, poly(butylene terephthalate) (PBT); PBT with COOH and OH end groups was thermally treated with DGEBA through extrusion in the presence of zinc acetylacetonate (Zn(acac)_2_), during which branching and cross-linking formation progressed [42]. The final cross-linked products possessed a PBT-based framework with OH groups in the network, exhibiting vitrimeric properties.

Goosens and Heuts et al. have also reported another simpler vitrimerization method of PBT by reacting it with glycerol in the presence of the trans-esterification catalyst, Zn(acac)_2_, via solid-state polymerization (Figure 8b) [43]. The incorporation of glycerol cross-linked the amorphous phase of PBT and provided OH groups in the network structure. The obtained PBT/glycerol materials showed stable rubbery plateaus up to a temperature above the melting temperature of PBT, whereas great stress–relaxation was observed at high temperatures, which indicated vitrimerization. The incorporation ratio of glycerol affected the relaxation rate owing to the change in the cross-link density. The fractured samples were recycled by hot pressing, and their mechanical properties evaluated by the dynamic mechanical thermal analysis were sustained after several recycling procedures.

Owing to the abovementioned useful properties of vitrimers, Terentjev and Ji et al. have reported the preparation of polyester-based vitrimer liquid crystalline elastomers (LCEs) with a trans-esterification-based bond exchange mechanism [44]. The biggest achievement in their research was the easy monodomain orientation of the liquid crystalline structure by making use of the network alternation property. In general, a monodomain orientation is required to attain a large reversible actuation for LCEs, while the orientation process has been regarded as a laborious step in conventional LCEs. In their method, cross-linked vitrimer LCEs were first stretched to achieve the orientation of mesogens; then, the temperature was made higher than the bond exchange temperature and the stretching was kept constant, which allowed the progress of the reconfiguration of the LC network into the monodomain orientation. This facile monodomain orientation can contribute to the practical application of LCEs. Similarly, Yu et al. have reported the preparation of vitrimer LCEs with a capability of facile monodomain orientation by simply reacting diacrylate mesogen, di-functional thiol spacer, and tetra-functional thiol cross-linkers via the Michael addition reaction in the presence of a catalyst, triazobicyclodecene (TBD) [45].

**Figure 9:**
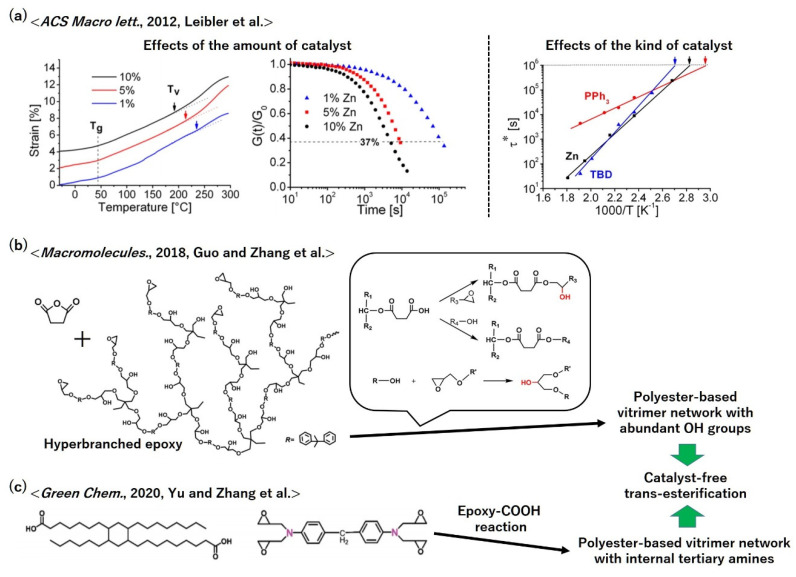
(**a**) Catalytic control over vitrimeric properties according to ref. [24]. Adapted with permission from ref. [24]. Copyright (2012) American Chemical Society. (**b**), (**c**) Examples of molecular designs of catalyst-free vitrimer polyesters according to (**b**) ref. [48] and (**c**) ref. [51]. (**b**) Adapted with permission from ref. [48]. Copyright (2018) American Chemical Society. (**c**) Adapted with permission from ref. [51], Copyright (2020) The Royal Society of Chemistry; permission conveyed through Copyright Clearance Center, Inc.

Polyester-based vitrimers with the trans-esterification bond exchange mechanism have an advantage in the precise tunability of vitrimeric properties, including the activation temperature of the bond exchange (vitrimer temperature, *T*_V_), relaxation rate, and activation energy, by varying the kind and amount of the catalysts [24,46,47]. The examples demonstrated by Leibler et al. are shown in Figure 9a [24]. In their research, the amount of catalyst, Zn(OAc)_2_, was first varied as 1, 5, and 10 mol% to the COOH groups in the feed, which resulted in a decrease in *T*_V_ and an increase in the bond exchange rate with an increase in the amount of the catalyst. Subsequently, using three different trans-estefication catalysts (i.e., Zn(OAc)_2_, TBD, and triphenylphosphine (PPh_3_)), they demonstrated control over the vitrimeric properties [24]. In addition, recently, trans-esterification-based polyester vitrimers have been found to be operated without adding bond exchange catalysts, by incorporating abundant free OH groups that served as both reacting moieties and catalysts [48,49,50]. As an example, Figure 9b represents the molecular design reported by Guo and Zhang et al. [48]. As another example of catalyst-free polyester vitrimers with a trans-esterification mechanism, tertiary amine groups were incorporated as internal catalysts to activate the trans-esterification in the network [51,52]; the molecular design reported by Yu and Zhang et al. is illustrated in Figure 9c [51]. These ideas are meaningful for practical application because the catalysts used in vitrimers are often toxic and may also cause deterioration after many recycling procedures.

### 5.2. Polylactide

**Figure 10:**
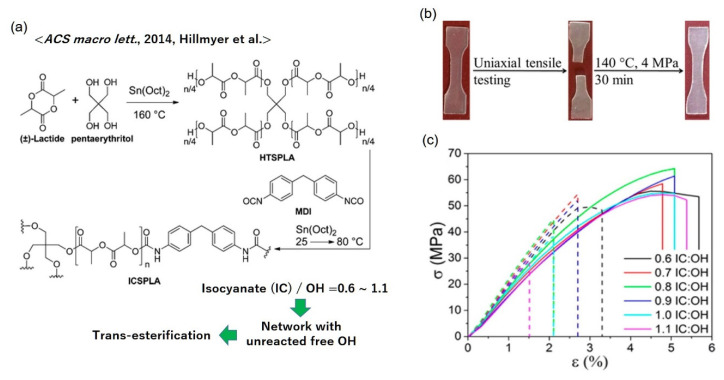
(**a**) Molecular design of polylactide vitrimers according to ref. [53]. The visual representation of healing and the healing level are shown in (**b**) and (**c**), respectively. In (**c**), the stress –strain curves (*σ* vs. *ε*) are summarized for the pristine (solid lines) and healed (broken lines) samples, where the IC:OH represents the ratio of isocyanate (IC) and OH groups in the feed. Adapted with permission from ref. [53]. Copyright (2014) American Chemical Society.

Polylactides (PLAs) are among the most studied bio-based thermoplastics; thus, the vitrimerization of PLAs is truly valuable in terms of the realization of a sustainable society. Hillmyer et al. have achieved the preparation of vitrimer PLAs with a trans-esterification-based bond exchange mechanism by cross-linking four-arm star-shaped poly((±)-lactide) (HTSPLA) with methylenediphenyl diisocyanate (MDI), where some OH groups were designed to remain unreacted for the activation of trans-esterification (Figure 10a) [53]. Stannous octoate (Sn(oct)_2_) was incorporated as the trans-esterification catalyst. Vitrimer PLAs showed excellent healing by compression molding (Figure 10b). In their design, the cross-link density, which was accompanied by the change in the OH group concentration, was tunable by varying the feed ratio of HTSPLA and MDI. Thus, they have investigated the effect of the cross-link density and OH concentration on the stress–relaxation rate and healing efficiency (Figure 10c), which revealed that a more loosely cross-linked network with higher mobility and higher OH concentration enabled a more efficient bond exchange.

### 5.3. Polycarbonates

**Figure 11:**
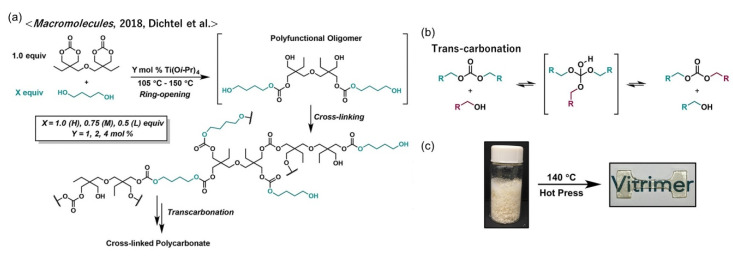
(**a**) Molecular design of vitrimer polycarbonates according to ref. [54]. The scheme of trans-carbonation is shown in (**b**). The visual representation of recyclability is shown in (**c**). Adapted with permission from ref. [54]. Copyright (2018) American Chemical Society.

Similar to trans-esterification, carbonates can undergo an exchange reaction, so-called trans-carbonation, in which a hydroxyl nucleophile reacts with a carbonate by forming an associative intermediate, followed by the release of the exchanged carbonate and hydroxyl group. The vitrimerization of polycarbonates (PCs) has an important meaning for sustainability because carbonates can be synthesized from CO_2_. Dichtel et al. prepared hydroxyl functionalized PC networks containing titanium isopropoxide, Ti(Oi-Pr)_4_, by reacting bis(6-membered cyclic carbonate) (bCC) and 1,4-butanediol (BD) (Figure 11a) [54]. The obtained PC exhibited vitrimeric properties, e.g., great stress–relaxation and an Arrhenius relationship of *τ* and *T*, and the relaxation rate varied depending on the feed ratio of bCC and BD, due to the changes in the cross-link density and OH concentration. Owing to the bond exchangeable nature (see the exchange mechanism in Figure 11b), the vitrimer PC attained efficient recyclability by hot pressing (Figure 11c). Moreover, they demonstrated the hydrolyzation and decarboxylation of a vitrimer PC in an aqueous acid, and the recollection of a clean monomer precursor by extraction with an organic solvent.

### 5.4. Polydimethylsiloxanes

**Figure 12:**
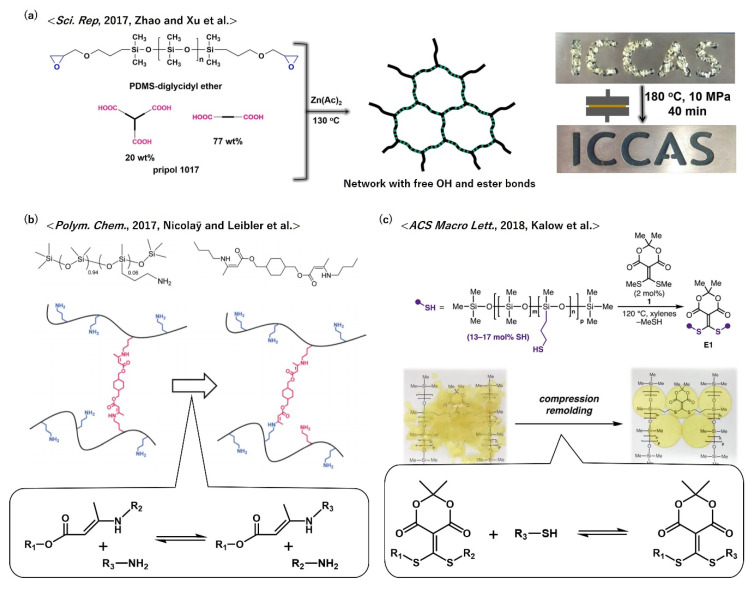
Molecular designs of vitrimer polydimethylsiloxanes according to (**a**) ref. [55], (**b**) ref. [56] (**b**), and (**c**) ref. [58]. In (**b**) and (**c**), the bond exchange mechanism of vinylogous urethane exchange and the trans-thioetherification of Meldrum’s acids are shown, respectively. (**a**) Adapted from ref. [55]. Publisher: Springer Nature, copyright © (2017), Springer Nature. (**b**) Adapted with permission from ref. [56], copyright (2017), The Royal Society of Chemistry; permission conveyed through Copyright Clearance Center, Inc. (**c**) Adapted with permission from ref. [58]. Copyright (2018) American Chemical Society.

Polydimethylsiloxanes (PDMSs) are also important industrial polymers due to their high transparency and usability as flexible elastomers in low temperature environments. As shown in the above examples of vitrimer polyesters and polycarbonates, trans-esterification bond exchange is applicable to the network of PDMSs, which may be the most simple approach for the vitrimerization of PDMSs. Zhao and Xu et al. reported vitrimer PDMS using telechelic PDMS-bearing epoxy groups at the end groups (PDMS-diglycidyl ether) (Figure 12a) [55]. PDMS-diglycidyl ether was reacted with Pripol 1017 ((a mixture of di-acid (23%) and tri-acid (77%)) in the presence of zinc acetate, which formed the PDMS-based network containing ester and OH groups near the junction point. Although the fraction of reactive groups, i.e., ester and OH groups, for the trans-esterification was much lower than that in the abovementioned cases of vitrimer polyesters and polycarbonates, the obtained PDMS elastomers showed vitrimeric properties. Simple hot pressing for the fractured pieces enabled multiple recycling of vitrimer PDMS.

As an alternative design using another bond exchange mechanism, Nicolaÿ and Leibler et al. successfully prepared vitrimer PDMS using the trans-amination of vinylogous urethane cross-links (Figure 12b) [56]. Vinylogous urethane exchange, which was first reported by Winne and Du Prez et al. [57], is an attractive associative exchange reaction, because the exchange reaction can efficiently progress without catalysts. PDMS with NH_2_ reactive side groups, i.e., poly[dimethylsiloxane-*co*-(3-aminopropyl)methylsiloxane] copolymer (PDMS-NH_2_), was mixed with a bis-vinylogous urethane compound to partially cross-link the PDMS chains. At high temperatures, the exchange reaction between the remaining free NH_2_ and vinylogous urethane progressed without exchange catalysts. By further reacting the free NH_2_ groups in the network with methyl acetoacetate, the relaxation rate was dramatically slowed down, which demonstrated the vitrimeric character quenching.

Another exchange reaction can be incorporated to prepare vitrimer PDMS. Kalow et al. reported the preparation of vitrimer PDMS using a reversible conjugate addition–elimination reaction with a Meldrum’s acid alkylidene that can be also operated without exchange catalysts (Figure 12c) [58]. The component PDMS with thiol side groups was transformed into vitrimers by reacting a fraction of the thiol groups with a Meldrum’s acid alkylidene derivative.

### 5.5. Polydienes

**Figure 13:**
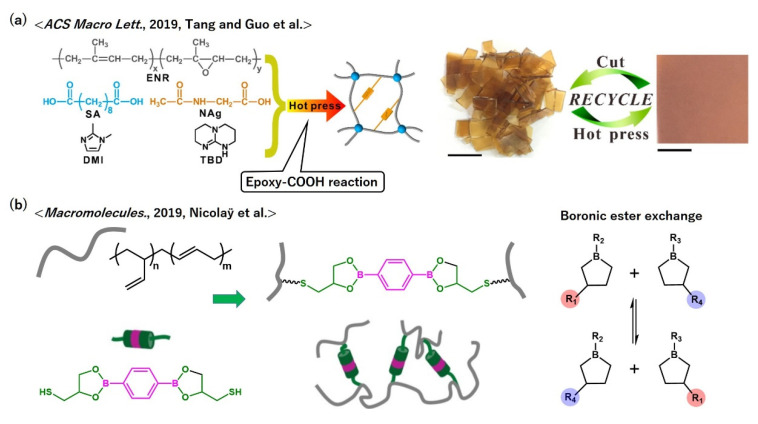
Molecular designs of vitrimer polydienes according to (**a**) ref. [59] and (**b**) ref. [60]. In (**a**), the visual representation of recyclability is also provided. In (**b**), the bond exchange mechanism of the boronic ester exchange is shown. (**a**) Adapted with permission from ref. [59]. Copyright (2019) American Chemical Society. (**b**) Adapted with permission from ref. [60]. Copyright (2019) American Chemical Society.

Polydienes are typically used to produce synthetic rubbers; thus, the vitrimerization of polydienes can be helpful to create novel functional rubbers. Tang and Guo et al. used commercial epoxidized polyisoprenes (Epoxy-ENR) and cross-linked Epoxy-ENR with a di-acid compound (sebacic acid, SA) in the presence of a trans-esterification catalyst, TBD (Figure 13a) [59]. *β*-Hydroxyl ester linages were generated by the epoxy-COOH reaction; thus, the final cross-linked materials behaved as vitrimers. They also revealed that the incorporation of additional hydrogen bonded cross-links, which were generated by the reaction of epoxy with N-acetylglycine (NAg), did not deteriorate the trans-esterification, while it contributed to enhance the tensile properties at room temperature because the hydrogen bonded cross-links acted as sacrificial bonds that could dissipate internal stress during elongation.

In addition to polyisoprenes, polybutadienes were transformed into vitrimers using dioxaborolane chemistry, which was reported by Nicolaÿ et al. (Figure 13b) [60]. They cross-linked polybutadiene chains by bis-thiol dioxaborolane molecules via the radical reaction. Dioxaborolane exchange, the so-called boronic ester exchange, has an associative bond exchange nature; thus, the resulting cross-link materials exhibited vitrimeric functions, e.g., great stress–relaxation while keeping a stable rubbery plateau, which eventually resulted in efficient multiple recyclability without impairing the elastomeric properties.

### 5.6. Polyurethanes

**Figure 14:**
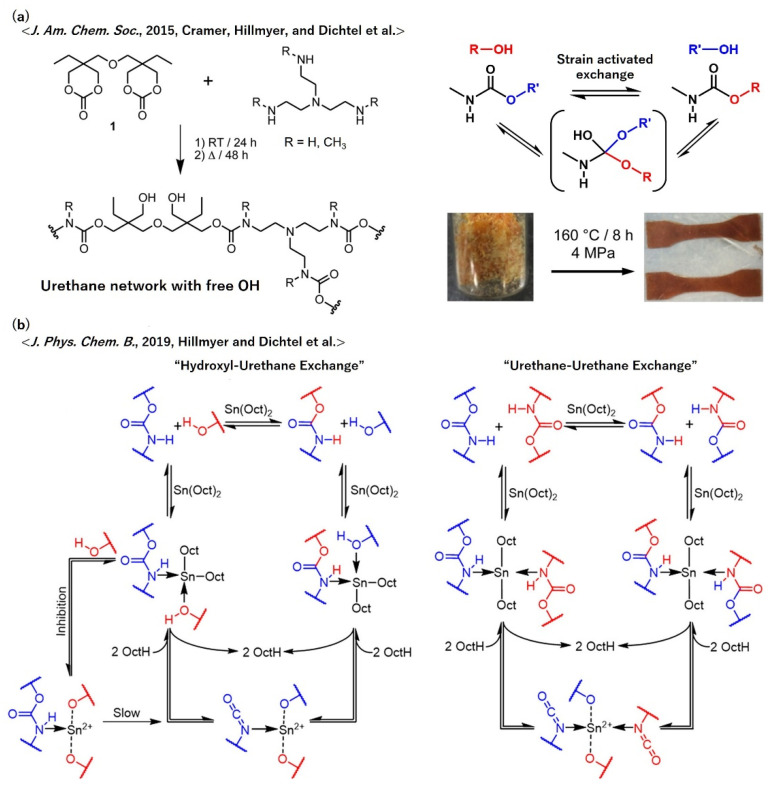
(**a**) Molecular design of vitrimer polyurethane according to ref. [61], where the mechanism of transcarbomoylation and a visual representation of recyclability are also shown. (**b**) Proposed mechanism of urethane reversion depending on the presence or absence of the metal catalyst, stannous octoate (Sn(Oct)_2_), according to ref. [63]. (**a**) Adapted with permission from ref. [61]. Copyright (2015) American Chemical Society. (**b**) Reprinted with permission from ref. [63]. Copyright (2019) American Chemical Society.

Polyurethanes (PUs) are suitable for various applications, depending on the component monomers, for example, as forms, elastomers, and coatings. Therefore, the vitrimerization of PUs has a considerable potential to add new functions to these products, such as the enhancement of thermal/mechanical toughness, while keeping re-processability and recyclability. Cramer, Hillmyer, and Dichtel et al. reported the preparation of a vitrimer PU using bis(cyclic carbonate) and tris(2-aminoethyl)amine as component monomers (Figure 14a) [61]. The obtained cross-linked PU, which bore urethane bonds and OH groups in the network structure, showed the vitrimeric properties, including great stress–relaxation and recyclability, simply by heating without adding catalysts for the bond exchange. They have investigated the bond exchange mechanism in detail, and demonstrated that the predominant bond exchange mechanism was not a reversion of carbamate to isocyanate, but transcarbomoylation that underwent an associative intermediate. More importantly, the DFT calculation revealed that the transcarbomoylation was accelerated by the mechanical torsional strain of the carbamate bond. Later, they also reported the effects of the network structure, especially in terms of the cross-link density on the reprocessability and stress–relaxation using similar polymer frameworks [62].

It should be here noted that the exchange reaction in the urethane network is somewhat complicated, as later pointed out by the same group, i.e., Hillmyer and Dichtel et al. Using model compounds, they revealed that urethane reversion, which was actually the dissociative exchange reaction, was probably the predominant exchange reaction that occurred in cross-linked polyurethane-based polymers. In addition, urethane reversion has two pathways, depending on the presence or absence of exogenous alcohol (Figure 14b). In the presence of exogenous alcohol, the exchange reaction (“hydroxyl-urethane exchange”), consisting of a urethane reversion and reaction with a new hydroxyl, is the predominant pathway, whereas in the absence of exogenous alcohol, the exchange reaction (“urethane-urethane exchange”), consisting of urethane reversion and recombination, is the predominant pathway. In both cases, the binding of OH or NH groups with the metal catalyst plays an important role in governing the exchange reaction mechanism [63,64]. Thus, the classification of polyurethane-based dynamic cross-linked materials into either true vitrimers or vitrimer-like materials has to be treated carefully.

### 5.7. Polyolefins

**Figure 15:**
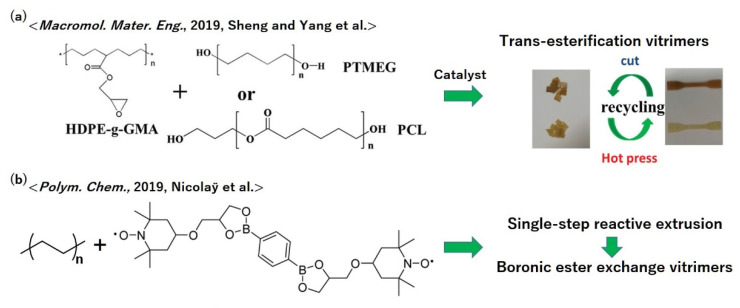
Molecular designs of vitrimer polyethylenes according to (**a**) ref. [65] and (**b**) ref. [66]. Please also see the bond exchange mechanism of boronic ester exchange in Figure 13. (**a**) Reprinted with permission from ref. [65]. Copyright (2019) John Wiley & Sons, Inc.; permission conveyed through Copyright Clearance Center, Inc. (**b**) Adapted with permission from ref. [66], Copyright (2019) The Royal Society of Chemistry; permission conveyed through Copyright Clearance Center, Inc.

Although the vast majority of plastic consumption in the world has been made from polyolefins, the vitrimerization of polyolefins has been a challenging issue, probably due to the difficulty in attaching reactive groups at multiple points along the chain. The simplest idea for vitrimerization may be the melt grafting of epoxy monomers into polyethylene chains, as reported by Sheng and Yang et al. (Figure 15a) [65]. Polyethylenes bearing glycidyl side groups were cross-linked by the reaction with a telechelic OH-terminated cross-links in the presence of the trans-esterification catalyst, which resulted in the formation of a network bearing *β*-hydroxyl esters. The obtained materials exhibited vitrimeric characters, and also recyclability by hot pressing.

As another idea for the vitrimerization of polyethylene, Nicolaÿ et al. demonstrated the incorporation of boronic ester exchange into a polyethylene-based network by single-step reactive extrusion (Figure 15b) [66]. The key for the synthesis is to use nitroxide radical grafting into polyethylene chains, where nitroxides are highly reactive toward carbon-centered radicals. A bis-boronic ester bearing two nitroxide functions was synthesized and reacted with component polyethylene, which resulted in the cross-linked polyethylene network. Owing to the occurrence of the associative bond exchange via boronic ester exchange, the obtained materials showed great stress–relaxation while keeping the stable rubbery plateau up to high temperatures above the melting point, which indicated the success of vitrimerization. This design has its advantage in the capability of associative bond exchange without adding catalysts.

### 5.8. Poly(meth)acrylates

**Figure 16:**
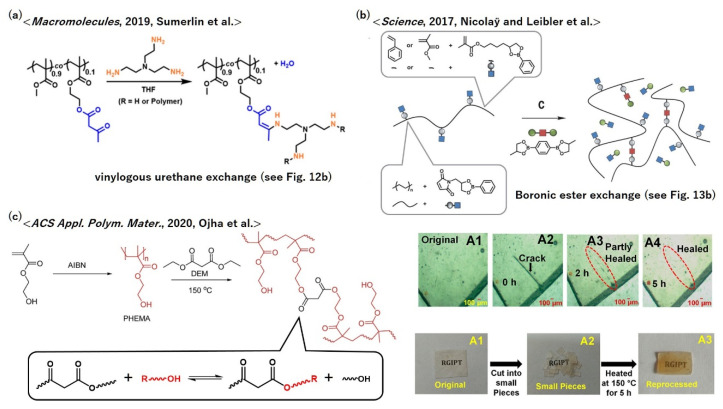
(**a**) Molecular designs of vitrimer poly(meth)acrylates according to (**a**) ref. [67], (**b**) ref. [68], and (**c**) ref. [69]. In (**c**), visual representations of scratch healability and recyclability are also shown, where the sample was heated at 150 °C for the healing test. (**a**) Adapted with permission from ref. [67]. Copyright (2019) American Chemical Society. (**b**) From (Rottger, M.; Domenech, T.; van der Weegen, R.; Breuillac, A.; Nicolaÿ, R.; Leibler, L, *Science*, 356 (6333), 62–65 (2017)). Reprinted with permission from AAAS. (**c**) Adapted with permission from ref. [69]. Copyright (2020) American Chemical Society.

The vitrimerization of poly(meth)acrylates has been explored because these are useful commercial polymers that are used as, e.g., acrylic resins, elastomers, and adhesive tapes. The simple incorporation of trans-esterification bond exchange is not suitable for the vitrimerization of poly(meth)acrylates, considering these polymers possess ester side groups and thus the elimination of side groups cannot be avoided during trans-esterification. Therefore, other bond exchange reactions have to be used for vitrimerization. As an alternative, Sumerlin et al. prepared poly(methyl methacrylate) (PMMA)-based vitrimers using a vinylogous urethane exchange (Figure 16a) [67]. First, they synthesized random copolymers of methyl methacrylate and (2-acetoacetoxy)ethyl methacrylate units, and the copolymer was cross-linked by tris(2-aminoethylamine) (TREN), where some fraction of amino groups remained unreacted. The cut pieces of vitrimer PMMA were merged into a disk- or bar-shaped sample by hot pressing, owing to the vinylogous urethane bond exchange.

Boronic ester metathesis, which was first introduced in Section 5.5, is another candidate for the vitrimerization of poly(meth)acrylates. Nicolaÿ and Leibler et al. designed and synthesized random copolymers of methyl methacrylate and another original methacrylate-bearing pendant dioxaborolane (Figure 16b) [68]. Then, the copolymers were cross-linked using a bisdioxaborolane. Bond exchange progressed via dioxaborolane metathesis, which provided the materials with vitrimeric characters. Note that they also demonstrated that the same strategy could be applied to polystyrene-based random copolymers.

In fact, there is one example of a vitrimer polymethacrylate with a trans-esterification based bond exchange mechanism (Figure 16c); Ojha et al. reported the preparation of such vitrimers using poly(hydroxyethyl methacrylate) (PHEMA). PHEMA was partially cross-linked by adding low-cost *β,β′*- diesters, i.e., diethyl malonate (DEM) [69]. In the cross-linked network, the trans-esterification of *β,β′*-diesters with pendant OH groups selectively occurred in the presence or absence of catalysts, where the ester side groups of the PHEMA chains did not participate in the exchange reaction. Therefore, in this method, the elimination of side groups did not occur, which shows the benefit of using *β,β′*-diesters in the simple vitrimerization of poly(meth)acrylates. The obtained materials exhibited crack healing and recyclability owing to the bond exchange at high temperatures.

### 5.9. Poly(styrene)

**Figure 17:**
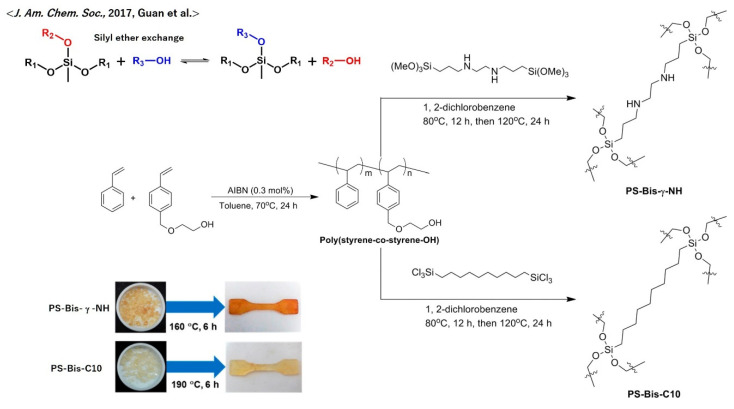
Molecular designs of vitrimer polystyrene according to ref. [70]. Please see the details of the cross-linking design in the text. Visual representations of the recyclability are also shown. Adapted with permission from ref. [70]. Copyright (2017) American Chemical Society.

The preparation of poly(styrene) (PS)-based vitrimers is actually very limited, although PS is one of the widely used polymers. Except for the above example of the vitrimerization of PS-based copolymers using dioxaborolane metathesis, according to Nicolaÿ and Leibler et al. (see Section 5.8), a good example of vitrimerization of PS was reported by Guan et al. (Figure 17) [70]. They incorporated silyl ether exchange in the network structure, consisting of a PS-based random copolymer bearing OH groups, poly(styrene-*co*-styrene-OH). To activate the silyl ether exchange reaction, sufficient amounts of free OH groups remained unreacted. The obtained cross-linked materials exhibited vitrimeric properties without catalysts, such as great stress–relaxation at high temperatures and recyclability by hot pressing. In addition, an acceleration of the exchange reaction was attained by introducing a neighboring amino moiety at the γ position, which was revealed by comparing the properties of PS-Bis-g-NH and PS-Bis-C10, shown in Figure 17.

### 5.10. Other Trends for the Practical Application of the Vitrimer Concept

In the above sections, various representative vitrimerization methodologies for industrial polymers were described. Of course, the vitrimerization of other non-classifiable polymers with unique functions, e.g., strong hydrophobicity or hydrophilicity, have been reported by several groups [71,72,73]. In addition to the abovementioned examples, there are recent trends in the vitrimer molecular designs. One trend is designed for block copolymer-based materials. As shown in the recent report from Epps and Sumerlin et al. [74] and Tang and Guo et al. [75], simple AB diblock copolymers can exhibit vitrimeric properties by incorporating associative bond exchange mechanisms in self-assembled A or B domains. Using this strategy, additional positive effects, such as the enhancement of creep resistance, can be attained, because non-vitrimeric domains have a considerably lower mobility than the vitrimeric block. A similar approach, i.e., the selective vitrimerization of a component block, is possible for multi-block copolymer systems [76]. In the above report from Epps and Sumerlin et al. [74], the investigation of a self-assembled structure has also been carried out by scattering experiments, and such a self-assembled structure was also validated for graft polymer-like vitrimers, according to Ricarte and Leibler et al. [77]. In their design, polyethylene chains (“backbone”) were linked through relatively long cross-linkers (dioxaborane maleimide, “graft”) having a repulsive interaction with the polyethylene. This pair of components self-assembled into graft-rich/poor domains at the mesoscale, in which the graft/cross-links formed smaller aggregations at the nanoscale.

In addition, the mechanical property modification/enhancement of vitrimer materials has also attracted great attention for practical applications. A typical example is the preparation of composite vitrimers. Thus far, various composite vitrimers have been demonstrated using different filler additives, such as carbon nanodots [78], carbon black [79], graphene [80], silica nanoparticles [81,82], Al_2_O_3_ [83], gold nanoparticles [84], cellulose nanofibers [85], carbon fibers [86], glass fibers [87,88], etc. [89,90,91]. In these reports, the functional groups on the filler surface were important parameters for vitrimeric property tuning, as well as for the good distribution of fillers in the vitrimer matrix. The second major approach for modification/enhancement is to use dual-type cross-links. In general, the mechanical properties at room temperature, such as Young’s modulus, elongation at break, and toughness, can be enhanced by incorporating sacrificial bonds, which is also applicable to vitrimer materials. For example, Tang and Guo et al. achieved an enhancement in the tensile properties of vitrimer elastomers by incorporating hydrogen-bonded sacrificial bonds into the vitrimer network, as described in the previous section [59] (see Section 5.5). In addition to the mechanical properties at room temperature, vitrimeric properties at high temperatures can be modified using dual-type cross-links, such as dynamic exchange bond + permanent bond [28,92] and dynamic exchange bond + another dynamic exchange bond [93].

## 6. Summary

As described in the above sections, vitrimers possess various useful functions, which are all attributed to the associative bond exchangeable nature. Currently, various molecular designs, on the basis of the commercial polymer frameworks, are available using different compatible bond exchange mechanisms, which provide several advantages for cross-linked materials, such as re-processability, healing ability, and recyclability. In addition, recent articles have demonstrated that the chemical recycling of cross-linked materials is possible, utilizing the bond exchange concept; by immersing cross-linked samples in suitable reactive solvents and applying heat, the bond exchange between the reactive groups in the network and the solvents was induced [94,95,96,97]. Thus, cross-linked materials were gradually de-polymerized during the bond exchange. In addition, to solve the problem of the depletion of petroleum resources, the use of bio-based monomers for the preparation of vitrimers has gained attention [98,99,100]. For the practical application of the vitrimer concept, the molecular-level control of the basic physics and flow properties of vitrimers, e.g., stress–relaxation and linear viscoelasticity, have also been studied, because these properties are closely related to material processing [101,102,103,104,105,106,107]. Because vitrimers are still relatively new, further development of vitrimers, including the design, the modification of properties, functionalization, and the discovery of new application ranges (such as for shape memory polymers [65,80,98,103,108,109,110,111,112,113] and 3D printer resin [114,115]), is highly favorable and can surely lead to the realization of a sustainable society.
